# *c*-5-Hy­droxy-*r*-2,*c*-4-bis­(meth­oxy­carbon­yl)-*t*-5-methyl-*t*-3-(3-nitro­phen­yl)cyclo­hexa­none

**DOI:** 10.1107/S1600536812027377

**Published:** 2012-06-27

**Authors:** S. Rizwana Begum, R. Hema, K. Pandiarajan, Sridhar Balasubramanian, A.G. Anitha

**Affiliations:** a Department of Physics, Seethalakshmi Ramaswami College (Autonomous), Tiruchirappalli 620 002, India; b Department of Chemistry, Annamalai University, Annamalai Nagar 608 002, India; c Laboratory of X-ray Crystallography, Indian Institute of Chemical Technology, Hyderabad 500 007, India

## Abstract

In the title compound, C_17_H_19_NO_8_ [systematic name = dimethyl 4-hydroxy-4-methyl-2-(3-nitrophenyl)-6-oxocyclohexane-1,3-dicarboxylate], the cyclo­hexa­none ring exhibits a chair conformation. The meth­oxy­carbonyl groups are oriented in opposite directions with respect to the cyclo­hexa­none ring. In the crystal, O—H⋯O hydrogen bonds links the mol­ecules into chains running parallel to the *a* axis. These chains are connected by weak C—H⋯O hydrogen bonds, forming sheets parallel to the *ab* plane.

## Related literature

For the pharmacological activity of cyclo­hexa­none derivatives, see: Puetz *et al.*(2003 ▶); Danyi *et al.* (1989 ▶); For related structure, see: Hema *et al.* (2006 ▶). For conformational analysis, see: Allinger (1977 ▶); Cremer & Pople (1975 ▶). For graph-set analysis, see: Bernstein *et al.* (1995 ▶).
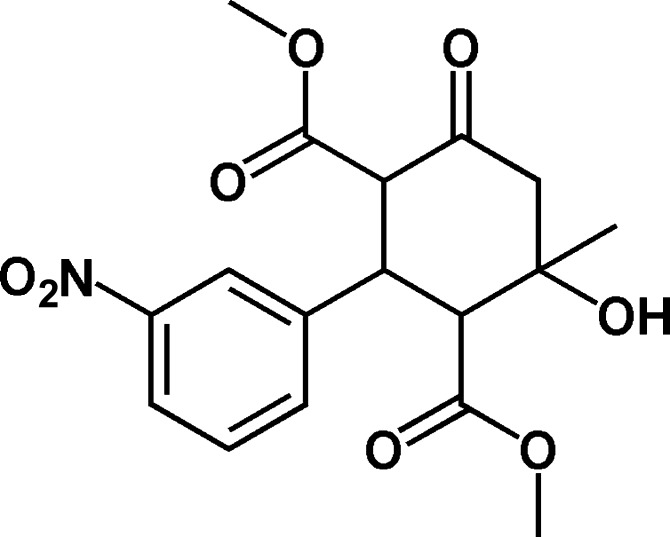


## Experimental

### 

#### Crystal data

C_17_H_19_NO_8_
*M* *_r_* = 365.33
Monoclinic, 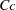

*a* = 20.1842 (18) Å
*b* = 5.7380 (5) Å
*c* = 15.5771 (14) Å
β = 108.357 (1)°
*V* = 1712.3 (3) Å^3^
*Z* = 4
Mo *K*α radiation
μ = 0.11 mm^−1^
*T* = 273 K
0.3 × 0.18 × 0.15 mm

#### Data collection

Bruker SMART CCD area-detector diffractometer
7818 measured reflections
1513 independent reflections
1469 reflections with *I* > 2σ(*I*)
*R*_int_ = 0.028

#### Refinement

*R*[*F* ^2^ > 2σ(*F* ^2^)] = 0.044
*wR*(*F* ^2^) = 0.112
*S* = 1.17
1513 reflections
239 parameters
2 restraints
H-atom parameters constrained
Δρ_max_ = 0.27 e Å^−3^
Δρ_min_ = −0.15 e Å^−3^

### 

Data collection: *SMART* (Bruker, 2001 ▶); cell refinement: *SAINT* (Bruker, 2001 ▶); data reduction: *SAINT*; program(s) used to solve structure: *SIR92* (Altomare *et al.*, 1994 ▶); program(s) used to refine structure: *SHELXL97* (Sheldrick, 2008 ▶); molecular graphics: *ORTEP-3* (Farrugia, 1999 ▶); software used to prepare material for publication: *SHELXL97* and *PLATON* (Spek, 2009 ▶).

## Supplementary Material

Crystal structure: contains datablock(s) I, global. DOI: 10.1107/S1600536812027377/go2056sup1.cif

Structure factors: contains datablock(s) I. DOI: 10.1107/S1600536812027377/go2056Isup2.hkl

Supplementary material file. DOI: 10.1107/S1600536812027377/go2056Isup3.cml

Additional supplementary materials:  crystallographic information; 3D view; checkCIF report

## Figures and Tables

**Table 1:** Hydrogen-bond geometry (Å, °)

*D*—H⋯*A*	*D*—H	H⋯*A*	*D*⋯*A*	*D*—H⋯*A*
C4—H4⋯O2^i^	0.98	2.46	3.374 (5)	154
C36—H36⋯O2^i^	0.93	2.51	3.414 (5)	164
O8—H8⋯O5^ii^	0.82	2.22	2.969 (5)	152

Symmetry codes: (i) 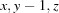; (ii) 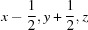.

**Fig. 1.**
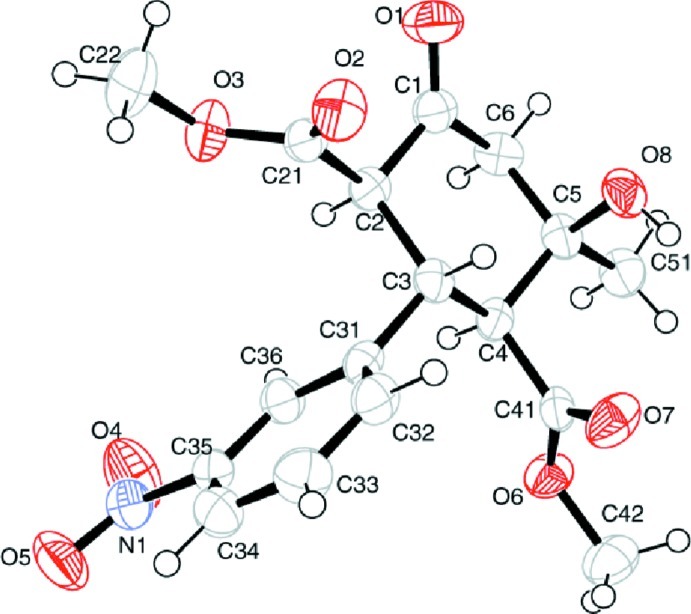
View of the molecule of the title compound showing the atom-labelling scheme. Displacement ellipsoids are drawn at the 50% probability level. H atoms are represented by circles of arbitrary radii.

**Fig. 2.**
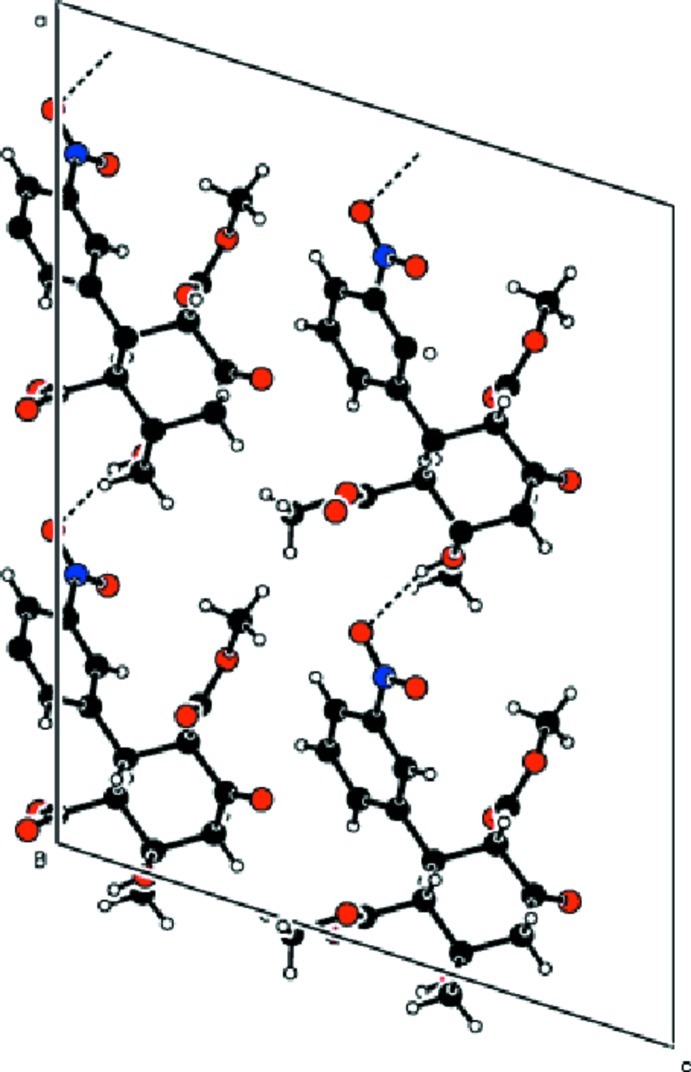
The O8–H8···O5 CHAINS, viewed along b axis. Dashed lines indicate hydrogen bonds.

## supplementary crystallographic information

### Comment

Cyclohexanone derivatives have potent pharmacological activity in the treatment of a broad spectrum of medical conditions(Puetz *et al.*, 2003). Cyclohexanone derivatives penetrate into the stratum corneum and alter the skin permeability of indomethacin by fluidizing or modifying the hard hydrophobic barrier of the corneum(Danyi *et al.*, 1989), thus giving an alternative method of administration of this compound which can cause serious gastric upsets.

In C_17_H_19_NO_8_, (I), (Fig. 1), the cyclohexanone ring adopts a chair conformation [Q=0.589 (4) Å, θ=173.0 (4)° and φ=16 (3)° (Cremer & Pople, 1975)]. The mean value [56.55 (16)°] of the endocyclic torsion angles of the cyclohexanone ring in (I) shows that it is slightly more puckered than the idealized cyclohexanone ring [54.1 (3)°, *MM2* calculation; (Allinger, 1977)]. The two methoxycarbonyl groups at C2 (C4—C3—C2—C21=-178.4 (2)°) and at C4 (C2—C3—C4—C41=-179.2 (3)°) are substituted in β-equatorial positions. The nitrophenyl ring(ring A) attached to C3 adopts an α equatorial orientation. The methyl and hydroxyl groups at C5 are oriented in β axial and equatorial positions respectively. The mean planes through C1, C3, C4 and C6 and ring A make a dihedral angle of 82.06 (12)°. This value is greater than that reported for a similar structure (73.76°) (Hema *et al.*, 2006). The dihedral angle between ring A and the carboxy groups O2–C21–O3 and O6–C41–O4 are 61.14 (24)° and 74.71 (27)°, respectively. These two carbonyl groups in (I) are twisted in opposite direction with C5–C4–C41–O7 and C1–C2–C21–O2 torsion angles of 75.8 (5)° and -73.0 (4)°, respectively.

The hydroxyl group forms a strong intermolecular hydrogen bond, O8–H8···O5(-0.5+x,0.5+y,z) linking the molecules into C(10) chains, (Bernstein *et al.*, 1995), Table 1 and Figure 2. The weak hydrogen bonds C4-H2···O2(x,-1+y,z) and C36-H36···O2(x,-1+y,z) link these chains into sheets which lie parallel to the*ab*-plane.

### Experimental

A mixture of methyl acetoacetate(11.6 g, 100 mmol), 3-nitrobenzaldehyde(7.55 g, 50 mmol) and methylamine(1.55 g, 50 mmol) in ethanol(50 ml) was heated to boiling. The reaction mixture was allowed to stand overnight. The separated solid was filtered and purified by recrystallization from ethanol to yield the title compound (yield 11.2 g, 75%, mp 475K).

### Refinement

H atoms were treated as riding atoms with C—H(aromatic), 0.93Å and C—H (aliphatic), 0.98Å with *U*_iso_ = 1.2Ueq(C) and C—H(methyl), 0.96Å, O—H, 0.82Å with *U*_iso_ = 1.5Ueq(). Friedel pairs were merged.

### Crystal data

**Table:**

C_17_H_19_NO_8_	*F*(000) = 768
*M**_r_* = 365.33	*D*_x_ = 1.417 Mg m^−^^3^
Monoclinic, *C**c*	Melting point: 475 K
Hall symbol: C -2yc	Mo *K*α radiation, λ = 0.71073 Å
*a* = 20.1842 (18) Å	Cell parameters from 2970 reflections
*b* = 5.7380 (5) Å	θ = 2.8–25°
*c* = 15.5771 (14) Å	µ = 0.11 mm^−^^1^
β = 108.357 (1)°	*T* = 273 K
*V* = 1712.3 (3) Å^3^	Prism, colourless
*Z* = 4	0.3 × 0.18 × 0.15 mm

### Data collection

**Table:**

Bruker SMART CCD area-detector diffractometer	1469 reflections with *I* > 2σ(*I*)
Radiation source: fine-focus sealed tube	*R*_int_ = 0.028
Graphite monochromator	θ_max_ = 25.0°, θ_min_ = 2.1°
ω scan	*h* = −24→24
7818 measured reflections	*k* = −6→6
1513 independent reflections	*l* = −18→18

### Refinement

**Table:**

Refinement on *F*^2^	Primary atom site location: structure-invariant direct methods
Least-squares matrix: full	Secondary atom site location: difference Fourier map
*R*[*F*^2^ > 2σ(*F*^2^)] = 0.044	Hydrogen site location: inferred from neighbouring sites
*wR*(*F*^2^) = 0.112	H-atom parameters constrained
*S* = 1.17	*w* = 1/[σ^2^(*F*_o_^2^) + (0.0657*P*)^2^ + 0.665*P*] where *P* = (*F*_o_^2^ + 2*F*_c_^2^)/3
1513 reflections	(Δ/σ)_max_ < 0.001
239 parameters	Δρ_max_ = 0.27 e Å^−^^3^
2 restraints	Δρ_min_ = −0.15 e Å^−^^3^

### Special details

**Table:**

Geometry. All e.s.d.'s (except the e.s.d. in the dihedral angle between two l.s. planes) are estimated using the full covariance matrix. The cell e.s.d.'s are taken into account individually in the estimation of e.s.d.'s in distances, angles and torsion angles; correlations between e.s.d.'s in cell parameters are only used when they are defined by crystal symmetry. An approximate (isotropic) treatment of cell e.s.d.'s is used for estimating e.s.d.'s involving l.s. planes.
Refinement. Refinement of *F*^2^ against ALL reflections. The weighted *R*-factor *wR* and goodness of fit *S* are based on *F*^2^, conventional *R*-factors *R* are based on *F*, with *F* set to zero for negative *F*^2^. The threshold expression of *F*^2^ > σ(*F*^2^) is used only for calculating *R*-factors(gt) *etc*. and is not relevant to the choice of reflections for refinement. *R*-factors based on *F*^2^ are statistically about twice as large as those based on *F*, and *R*- factors based on ALL data will be even larger.

### Fractional atomic coordinates and isotropic or equivalent isotropic displacement parameters (Å^2^)

**Table:**

	*x*	*y*	*z*	*U*_iso_*/*U*_eq_	
C3	0.12737 (18)	0.5989 (6)	0.1103 (2)	0.0308 (7)	
H3	0.0992	0.7412	0.0959	0.037*	
C2	0.17193 (17)	0.6084 (6)	0.2116 (2)	0.0316 (8)	
H2	0.2008	0.4675	0.2250	0.038*	
C1	0.12635 (19)	0.6086 (6)	0.2729 (2)	0.0370 (8)	
C6	0.0735 (2)	0.4139 (7)	0.2538 (3)	0.0400 (9)	
H61	0.0438	0.4325	0.2916	0.048*	
H62	0.0975	0.2657	0.2686	0.048*	
C5	0.02901 (18)	0.4137 (6)	0.1554 (3)	0.0348 (8)	
C4	0.07746 (18)	0.3893 (6)	0.0952 (2)	0.0311 (7)	
H4	0.1052	0.2467	0.1126	0.037*	
C31	0.17510 (18)	0.5939 (6)	0.0524 (2)	0.0312 (7)	
C32	0.1713 (2)	0.7685 (7)	−0.0109 (2)	0.0381 (8)	
H32	0.1375	0.8839	−0.0197	0.046*	
C33	0.2168 (2)	0.7735 (8)	−0.0610 (3)	0.0468 (10)	
H33	0.2127	0.8903	−0.1037	0.056*	
C34	0.2678 (2)	0.6082 (8)	−0.0482 (3)	0.0444 (9)	
H34	0.2992	0.6120	−0.0809	0.053*	
C35	0.27115 (19)	0.4360 (7)	0.0145 (3)	0.0380 (8)	
C36	0.22569 (18)	0.4240 (6)	0.0639 (2)	0.0361 (8)	
H36	0.2289	0.3029	0.1047	0.043*	
C41	0.03373 (17)	0.3699 (6)	−0.0021 (2)	0.0317 (7)	
C42	−0.0151 (3)	0.1008 (8)	−0.1194 (3)	0.0558 (11)	
H421	−0.0626	0.1288	−0.1220	0.084*	
H422	−0.0100	−0.0590	−0.1345	0.084*	
H423	−0.0032	0.2008	−0.1617	0.084*	
C21	0.22025 (18)	0.8146 (6)	0.2304 (2)	0.0335 (8)	
C22	0.3365 (2)	0.9385 (9)	0.2956 (4)	0.0607 (13)	
H221	0.3412	0.9999	0.2405	0.091*	
H222	0.3806	0.8782	0.3326	0.091*	
H223	0.3217	1.0603	0.3277	0.091*	
C51	−0.0253 (2)	0.2189 (7)	0.1379 (3)	0.0452 (9)	
H511	−0.0524	0.2361	0.1782	0.068*	
H512	−0.0021	0.0706	0.1479	0.068*	
H513	−0.0555	0.2278	0.0765	0.068*	
N1	0.32670 (18)	0.2619 (7)	0.0308 (2)	0.0492 (9)	
O1	0.13259 (17)	0.7525 (5)	0.3311 (2)	0.0556 (8)	
O2	0.20216 (15)	1.0111 (5)	0.2094 (2)	0.0485 (7)	
O3	0.28532 (14)	0.7538 (5)	0.2748 (2)	0.0472 (7)	
O7	0.00392 (16)	0.5267 (5)	−0.04872 (19)	0.0502 (7)	
O8	−0.00399 (14)	0.6359 (4)	0.14196 (19)	0.0415 (6)	
H8	−0.0331	0.6410	0.0915	0.062*	
O6	0.03014 (14)	0.1479 (4)	−0.03018 (19)	0.0434 (7)	
O4	0.32638 (19)	0.1005 (7)	0.0817 (3)	0.0780 (12)	
O5	0.37036 (18)	0.2827 (8)	−0.0070 (2)	0.0742 (12)	

### Atomic displacement parameters (Å^2^)

**Table:**

	*U*^11^	*U*^22^	*U*^33^	*U*^12^	*U*^13^	*U*^23^
C3	0.0308 (17)	0.0229 (15)	0.0379 (19)	0.0038 (13)	0.0098 (14)	0.0007 (14)
C2	0.0335 (18)	0.0236 (16)	0.0343 (19)	0.0063 (13)	0.0057 (15)	0.0026 (14)
C1	0.039 (2)	0.0347 (19)	0.035 (2)	0.0031 (16)	0.0090 (16)	0.0009 (17)
C6	0.050 (2)	0.0342 (19)	0.039 (2)	−0.0011 (16)	0.0178 (17)	0.0015 (15)
C5	0.0335 (18)	0.0222 (17)	0.050 (2)	−0.0015 (13)	0.0144 (17)	−0.0028 (14)
C4	0.0281 (16)	0.0228 (16)	0.0383 (19)	0.0020 (13)	0.0045 (14)	−0.0022 (14)
C31	0.0296 (16)	0.0269 (16)	0.0316 (17)	−0.0010 (13)	0.0017 (14)	−0.0028 (13)
C32	0.038 (2)	0.034 (2)	0.0384 (19)	0.0075 (15)	0.0061 (16)	0.0040 (15)
C33	0.054 (2)	0.049 (2)	0.038 (2)	0.0008 (19)	0.0149 (19)	0.0114 (17)
C34	0.039 (2)	0.056 (2)	0.041 (2)	−0.0011 (17)	0.0170 (17)	0.0022 (18)
C35	0.0299 (18)	0.044 (2)	0.0379 (19)	0.0031 (15)	0.0074 (15)	−0.0089 (16)
C36	0.0356 (19)	0.0345 (19)	0.0371 (19)	0.0040 (15)	0.0099 (16)	0.0010 (15)
C41	0.0226 (16)	0.0292 (17)	0.0418 (19)	0.0021 (13)	0.0081 (14)	−0.0025 (15)
C42	0.062 (3)	0.045 (2)	0.050 (3)	−0.0019 (19)	0.004 (2)	−0.012 (2)
C21	0.0342 (18)	0.0328 (19)	0.0324 (18)	0.0013 (15)	0.0090 (14)	−0.0007 (15)
C22	0.042 (2)	0.067 (3)	0.066 (3)	−0.017 (2)	0.007 (2)	0.006 (2)
C51	0.042 (2)	0.036 (2)	0.058 (3)	−0.0063 (16)	0.0160 (19)	−0.0048 (18)
N1	0.0377 (18)	0.060 (2)	0.047 (2)	0.0110 (16)	0.0095 (16)	−0.0060 (17)
O1	0.066 (2)	0.0561 (18)	0.0502 (17)	−0.0150 (15)	0.0260 (15)	−0.0196 (15)
O2	0.0460 (16)	0.0306 (15)	0.0640 (18)	0.0004 (11)	0.0102 (13)	0.0031 (13)
O3	0.0317 (13)	0.0435 (15)	0.0584 (17)	−0.0014 (11)	0.0027 (12)	0.0060 (13)
O7	0.0573 (17)	0.0346 (14)	0.0435 (15)	0.0095 (13)	−0.0060 (13)	0.0020 (12)
O8	0.0380 (14)	0.0319 (13)	0.0539 (16)	0.0078 (11)	0.0135 (12)	−0.0042 (12)
O6	0.0487 (16)	0.0309 (14)	0.0432 (14)	0.0031 (12)	0.0039 (12)	−0.0083 (11)
O4	0.066 (2)	0.067 (2)	0.109 (3)	0.0325 (18)	0.039 (2)	0.023 (2)
O5	0.0514 (19)	0.118 (3)	0.061 (2)	0.033 (2)	0.0295 (17)	0.005 (2)

### Geometric parameters (Å, º)

**Table:**

C3—C31	1.513 (5)	C34—C35	1.376 (6)
C3—C4	1.539 (5)	C34—H34	0.9300
C3—C2	1.551 (5)	C35—C36	1.372 (5)
C3—H3	0.9800	C35—N1	1.463 (5)
C2—C21	1.502 (5)	C36—H36	0.9300
C2—C1	1.521 (5)	C41—O7	1.193 (4)
C2—H2	0.9800	C41—O6	1.341 (4)
C1—O1	1.203 (4)	C42—O6	1.428 (5)
C1—C6	1.508 (5)	C42—H421	0.9600
C6—C5	1.513 (5)	C42—H422	0.9600
C6—H61	0.9700	C42—H423	0.9600
C6—H62	0.9700	C21—O2	1.199 (5)
C5—O8	1.423 (4)	C21—O3	1.324 (4)
C5—C51	1.529 (5)	C22—O3	1.444 (5)
C5—C4	1.559 (5)	C22—H221	0.9600
C4—C41	1.499 (5)	C22—H222	0.9600
C4—H4	0.9800	C22—H223	0.9600
C31—C36	1.382 (5)	C51—H511	0.9600
C31—C32	1.392 (5)	C51—H512	0.9600
C32—C33	1.378 (5)	C51—H513	0.9600
C32—H32	0.9300	N1—O5	1.209 (5)
C33—C34	1.368 (6)	N1—O4	1.221 (5)
C33—H33	0.9300	O8—H8	0.8200
			
C31—C3—C4	113.8 (3)	C32—C33—H33	119.7
C31—C3—C2	109.4 (3)	C33—C34—C35	117.9 (4)
C4—C3—C2	109.0 (3)	C33—C34—H34	121.1
C31—C3—H3	108.2	C35—C34—H34	121.1
C4—C3—H3	108.2	C36—C35—C34	122.8 (3)
C2—C3—H3	108.2	C36—C35—N1	118.7 (3)
C21—C2—C1	111.2 (3)	C34—C35—N1	118.5 (4)
C21—C2—C3	111.0 (3)	C35—C36—C31	119.3 (3)
C1—C2—C3	111.5 (3)	C35—C36—H36	120.3
C21—C2—H2	107.6	C31—C36—H36	120.3
C1—C2—H2	107.6	O7—C41—O6	123.5 (3)
C3—C2—H2	107.6	O7—C41—C4	125.7 (3)
O1—C1—C6	123.8 (3)	O6—C41—C4	110.8 (3)
O1—C1—C2	122.2 (3)	O6—C42—H421	109.5
C6—C1—C2	113.9 (3)	O6—C42—H422	109.5
C1—C6—C5	111.0 (3)	H421—C42—H422	109.5
C1—C6—H61	109.4	O6—C42—H423	109.5
C5—C6—H61	109.4	H421—C42—H423	109.5
C1—C6—H62	109.4	H422—C42—H423	109.5
C5—C6—H62	109.4	O2—C21—O3	123.9 (3)
H61—C6—H62	108.0	O2—C21—C2	124.4 (3)
O8—C5—C6	104.4 (3)	O3—C21—C2	111.8 (3)
O8—C5—C51	110.6 (3)	O3—C22—H221	109.5
C6—C5—C51	110.1 (3)	O3—C22—H222	109.5
O8—C5—C4	110.2 (3)	H221—C22—H222	109.5
C6—C5—C4	109.0 (3)	O3—C22—H223	109.5
C51—C5—C4	112.2 (3)	H221—C22—H223	109.5
C41—C4—C3	111.2 (3)	H222—C22—H223	109.5
C41—C4—C5	109.5 (3)	C5—C51—H511	109.5
C3—C4—C5	110.0 (3)	C5—C51—H512	109.5
C41—C4—H4	108.7	H511—C51—H512	109.5
C3—C4—H4	108.7	C5—C51—H513	109.5
C5—C4—H4	108.7	H511—C51—H513	109.5
C36—C31—C32	118.2 (3)	H512—C51—H513	109.5
C36—C31—C3	121.3 (3)	O5—N1—O4	123.0 (4)
C32—C31—C3	120.4 (3)	O5—N1—C35	119.0 (4)
C33—C32—C31	121.2 (3)	O4—N1—C35	118.0 (3)
C33—C32—H32	119.4	C21—O3—C22	116.5 (3)
C31—C32—H32	119.4	C5—O8—H8	109.5
C34—C33—C32	120.5 (4)	C41—O6—C42	116.7 (3)
C34—C33—H33	119.7		
			
C31—C3—C2—C21	56.6 (3)	C36—C31—C32—C33	−0.3 (5)
C4—C3—C2—C21	−178.4 (3)	C3—C31—C32—C33	176.6 (4)
C31—C3—C2—C1	−178.8 (3)	C31—C32—C33—C34	−1.1 (6)
C4—C3—C2—C1	−53.8 (4)	C32—C33—C34—C35	1.2 (6)
C21—C2—C1—O1	−3.4 (5)	C33—C34—C35—C36	0.1 (6)
C3—C2—C1—O1	−128.0 (4)	C33—C34—C35—N1	−178.3 (4)
C21—C2—C1—C6	176.5 (3)	C34—C35—C36—C31	−1.5 (6)
C3—C2—C1—C6	52.0 (4)	N1—C35—C36—C31	176.9 (3)
O1—C1—C6—C5	125.7 (4)	C32—C31—C36—C35	1.5 (5)
C2—C1—C6—C5	−54.2 (4)	C3—C31—C36—C35	−175.3 (3)
C1—C6—C5—O8	−59.9 (4)	C3—C4—C41—O7	−46.0 (5)
C1—C6—C5—C51	−178.7 (3)	C5—C4—C41—O7	75.8 (5)
C1—C6—C5—C4	57.9 (4)	C3—C4—C41—O6	136.2 (3)
C31—C3—C4—C41	−56.8 (4)	C5—C4—C41—O6	−102.0 (3)
C2—C3—C4—C41	−179.2 (3)	C1—C2—C21—O2	−72.9 (4)
C31—C3—C4—C5	−178.2 (3)	C3—C2—C21—O2	51.9 (5)
C2—C3—C4—C5	59.4 (3)	C1—C2—C21—O3	105.8 (3)
O8—C5—C4—C41	−70.4 (3)	C3—C2—C21—O3	−129.4 (3)
C6—C5—C4—C41	175.6 (3)	C36—C35—N1—O5	−173.9 (4)
C51—C5—C4—C41	53.3 (4)	C34—C35—N1—O5	4.6 (5)
O8—C5—C4—C3	52.0 (4)	C36—C35—N1—O4	6.6 (6)
C6—C5—C4—C3	−62.0 (4)	C34—C35—N1—O4	−174.9 (4)
C51—C5—C4—C3	175.8 (3)	O2—C21—O3—C22	−1.9 (6)
C4—C3—C31—C36	−65.9 (4)	C2—C21—O3—C22	179.4 (4)
C2—C3—C31—C36	56.3 (4)	O7—C41—O6—C42	−3.4 (5)
C4—C3—C31—C32	117.4 (3)	C4—C41—O6—C42	174.5 (3)
C2—C3—C31—C32	−120.5 (3)		

### Hydrogen-bond geometry (Å, º)

**Table:**

*D*—H···*A*	*D*—H	H···*A*	*D*···*A*	*D*—H···*A*
C4—H4···O2^i^	0.98	2.46	3.374 (5)	154
C36—H36···O2^i^	0.93	2.51	3.414 (5)	164
O8—H8···O5^ii^	0.82	2.22	2.969 (5)	152

Symmetry codes: (i) *x*, *y*−1, *z*; (ii) *x*−1/2, *y*+1/2, *z*.
